# Polypropylene/Basalt Fabric Laminates: Flexural Properties and Impact Damage Behavior

**DOI:** 10.3390/polym12051079

**Published:** 2020-05-08

**Authors:** Pietro Russo, Ilaria Papa, Vito Pagliarulo, Valentina Lopresto

**Affiliations:** 1 Institute for Polymers, Composites and Biomaterials, National Research Council, Via Campi Flegrei 34, 80078 Pozzuoli-Naples, Italy; pietro.russo@unina.it; 2 Department of Chemical, Materials and Production Engineering, University of Naples Federico II, P.le Vincenzo Tecchio 80, 80125 Naples, Italy; ilaria.papa@unina.it; 3 Institute of Applied Sciences & Intelligent Systems “E. Caianiello”, Via Campi Flegrei 34, 80078 Pozzuoli-Naples, Italy; v.pagliarulo@isasi.cnr.it

**Keywords:** polypropylene, basalt fibers, composite laminate, flexural, impact damage

## Abstract

Recently, the growing interests into the environmental matter are driving the research interest to the development of new eco-sustainable composite materials toward the replacement of synthetic reinforcing fibers with natural ones and exploiting the intrinsic recyclability of thermoplastic resins even for uses in which thermosetting matrices are well consolidated (e.g., naval and aeronautical fields). In this work, polypropylene/basalt fabric composite samples were prepared by film stacking and compression molding procedures. They have been studied in terms of flexural and low-velocity impact behavior. The influence related to the matrix modification with a pre-optimized amount of maleic anhydride grafted PP as coupling agent was studied. The mechanical performances of the composite systems were compared with those of laminates consisting of the pure matrix and obtained by hot-pressing of PP pellets and PP films used in the stacking procedure. Results, on one side, demonstrated a slight reduction of both static and dynamic parameters at the break for specimens from superimposed films to ones prepared from PP pellets. Moreover, an outstanding improvement of mechanical performances was shown in the presence of basalt layers, especially for compatibilized samples.

## 1. Introduction

The growing diffusion of plastics in all industrial fields and concerns related to their environmental impact and secure disposal at the end of the useful life has been the main reason motivating academic and industrial research towards the study and development of new eco-sustainable materials. So, an increasing interest in natural fibers as polymers reinforcement (conventional and bio-sources derived) has been detected. In this context, basalt fibers have gained outstanding attention as reinforcing fibers concerning traditional glass and carbon-based ones [1,2]. Specifically, the interest of the research toward the use of basalt fibers was driven by their remarkable properties such as relatively low cost, sustainability, enhanced mechanical properties, non-flammability, high chemical stability, excellent sound and thermal insulation [3]. Basalt fibers, derived from natural rocks in volcanic regions with technologies similar to that of glass fibers’ forming, are mainly composed of silica (SiO_2_) and alumina (Al_2_O_3_) [4] and have a melting temperature ranging from 1500 °C and 1700 °C with an average diameter between 9 and 13 micrometers. In the last decades, basalt fibers, included in thermosetting and thermoplastic resins for uses in several fields as transportation, defense and constructions, have largely demonstrated their potentiality as eco-sustainable substitutes of glass fibers, especially if an adequate adhesion at the interface with the surrounding polymer phase is ensured. 

Concerning thermosetting matrices, it is possible to obtain great benefits, primarily in terms of mechanical and thermal properties, including basalt fibers in epoxy, polyester and vinyl ester resins [5,6,7,8,9]. However—with the awareness that the sustainability of new products can be encouraged by the recyclability of the involved matrices—recently, the focus of the research has been increasingly devoted to thermoplastic systems mainly based on polypropylene [10,11,12], polyethene [13,14], polyamides [15,16,17,18], polyesters [19,20] as well as polymer blends [21] and hybrids [22,23]. 

In particular, polypropylene resins, primarily used for mass productions due to their low cost and full versatility in terms of processability and properties, still receive an extraordinary interest with research efforts mainly aimed to improve their performances further and extend their field of application more and more. 

In this regard, Szabo et al. [11] demonstrated that the mechanical properties of polypropylene composites packed with different short fibers are strongly affected by the content and direction of the same. As witnessed by scanning electron micrographs, samples showed damage mechanisms as fiber pull out in the perpendicular direction and debonding along the longitudinal direction depending on the manufacturing process used. 

Guo et al. [12] considered basalt fabric modified with the aid of a silane coupling agent KH550 to reinforce a commercial polypropylene resin. Hydrophilicity and lipophilicity tests of modified basalt fibers allowed a preliminary optimization of the pre-treatment parameters. Time and coupling agent content at values of 1.5 h and 6% by weight have been varied, respectively. The surface modification of basalt fibers significantly improved tensile, impact resistance, bending properties and thermal stability of the PP matrix giving rise to composites with better dynamic viscoelasticity compared to ones containing the same content of unmodified basalt fibers.

Greco et al. [24] highlighted the crucial role of structural features as the microcrystal size and the amorphous content of basalt fibers in determining their mechanical properties as well as the expected enhancement of mechanical performances of polypropylene composites by increasing the fiber-matrix interface goodness.

In the present paper, polypropylene/basalt fabric composite laminates, prepared by film stacking and hot-pressing procedures, were analyzed. Flexural and low-velocity impact properties were studied, taking pure PP specimens as reference materials and considering influences related to the matrix modification with a pre-optimized amount of a maleic anhydride grafted PP as a coupling agent. Mechanical results, also interpreted in light of indentation measurements, demonstrated that, although filming of the matrix does not significantly compromise mechanical performances of the PP matrix, the presence of basalt layers induces a marked improvement of composite performances. Moreover, the preliminary modification of the matrix to enhance the interfacial adhesion further improves the flexural performances, especially in terms of strength allowing to withstand higher impact loading concerning neat PP based composite laminates. Premised that for both investigated composites, no penetration seems to occur and the more significant matrix-reinforcement interaction for compatibilized composite laminamakesmake them less prone to plastic deformations as also evidenced by the indentation measurements. 

## 2. Experimental

### 2.1. Materials

In this paper a polypropylene matrix (Hyosung Topilene PP J640, MFI@230 °C, 2.16 kg: 10 g/10 min) provided by Songhan Plastic Technology Co. Ltd. (Shangai, China) and a plain wave basalt fabric (areal weight: 210 g/m^2^) from Incotelogy GmbH (Pulheim, Germany). The resin was used as received (PP) or pre-modified (PPC) by the inclusion of 2 wt.% of a coupling agent Polybond 3000 (MFI@190 °C, 2.16 kg: 400 g/10 min) from Chemtura (Philadelphia, Pennsylvania, USA). This latter is a polypropylene grafted with maleic anhydride (PP-*g*-MA) with 1.2 wt.% of MA. 

In more details, the modification of the commercial PP was performed with the aid of a co-rotating twin-screw extruder Collin Teach-Line ZK25T (Ebersberg, Germany), operating with the following temperature profile: 180–190–205–195–85 °C, from the hopper to the die, and at a screw speed of 60 rpm. The neat PP and the extruded pellets of modified PP were transformed in flat films with a thickness approximately equal to 35–40 μm using a Collin flat die extruder Teach-Line E20T equipped with a calendar CR72T (Ebersberg, Germany). For this stage, the processing was conducted at a screw speed of 55 rpm, setting the temperature profile along the screw at 180, 190, 200, 190 and 185 °C.

### 2.2. Laminates Preparation

**Figure 1:**
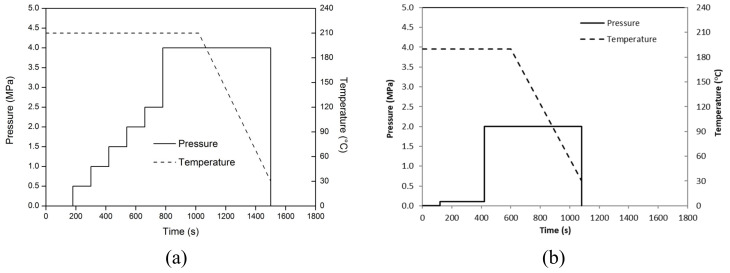
Hot-pressing conditions for (**a**) composite and reference (**b**) plates.

The conventional film stacking technique was used to obtain laminates. Layers of plastic films and basalt fabric, alternatively overlapped, are subjected to a pre-optimized pressure and temperature cycle (see Figure 1a) by press Collin GmbH (Edersberg, Germany) Mod. P400E. 

In this way, 380 mm × 380 mm plaques constituted by 16 plies, symmetrically settled with respect to the medium plane, with an average thickness of 2.6 mm and a volumetric content of reinforcement of about 50% (ASTM D 3171-04, Test Method II) have been produced. 

As a reference, plates of only PP matrix, with a thickness approximately equal to 2.5 mm, were prepared from PP granules and by superposition of plastic films mentioned above, according to the conditions shown in Figure 1b.

### 2.3. Experimental Techniques

#### 2.3.1. Static Mechanical Properties 

Flexural tests have been carried out by a universal Instron dynamometer Mod. 5564 equipped with a load cell of 1 kN. Specimens 12.7 mm wide and 100 mm long were cut from each sample laminate and loaded at room temperature in the three-point bending mode, according to the ASTM D790 standard, using a cross-head speed set at 2.5 mm/min and a span equal to 70 mm. 

Flexural parameters evaluated by processing typical stress–strain curves were averaged on at least 5 determination for each investigated sample. 

#### 2.3.2. Impact Properties 

Falling weight machine (Ceast Fractovis, Torino, Italy) was used for the impact tests at complete penetration, to obtain and study the full load–displacement curves to get useful information about the response of the laminates and for investigating the effect of the varied parameters. The penetration energy, U_p_, set to obtain the complete penetration of all composite systems studied is equal to 100 J. Then, different energy levels (U = 3, 8, 15 J) were chosen to carry out the so-called indentation tests, useful to study the damage start and evolution. The rectangular samples, 100 × 150 mm, cut by a diamond saw from the original panels, were supported by the clamping device suggested by the ASTM D7137 Standard and were loaded in the center by an instrumented cylindrical impactor with a hemispherical nose, 19.8 mm in diameter. Tests were carried out using an impactor (m = 3.640 kg) placed at specific heights to obtain the selected impact energies. After all the impact tests, the samples were observed by a visual 2D stylus profilometer (Dektak XT) to derive quantitative information about step heights, which is indentation depth along the impacted areas.

## 3. Results and Discussion

**Figure 2:**
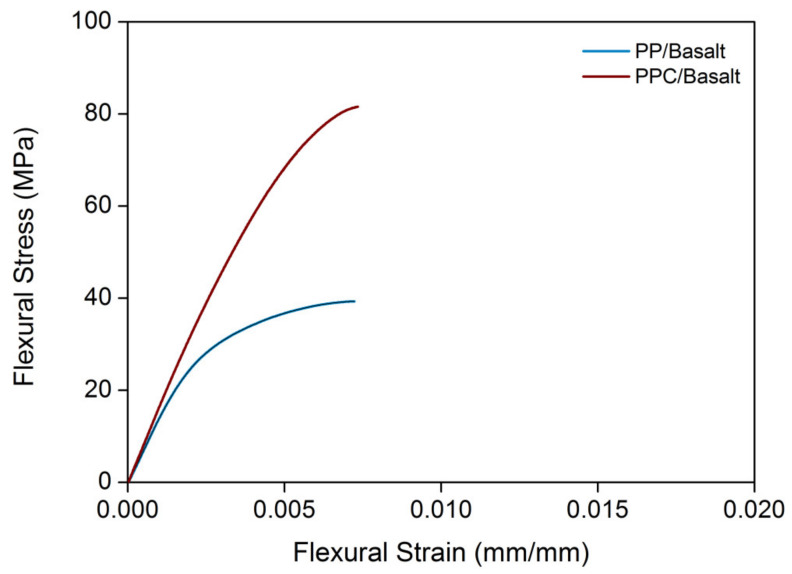
Flexural stress–strain curves.

The flexural stress–strain curves of composite specimens based on the neat and pre-modified film of polypropylene (coded as PP and PPC, respectively) are reported in Figure 2. At the same time, the evaluated mechanical parameters (modulus and strength) are summarized in Table 1. Flexural parameters compare quite favorably with those of other studies [5]. 

With regard to composite systems, previous morphological analyses conducted by electron scanning microscopy (SEM) on the same laminates have shown that for PP/Basalt composites, as expected given the nature of the phases involved, a poor interfacial adhesion is evident with images showing reinforcing fibers predominantly smooth and clean. On the contrary, for the PPC/basalt system, the authors reported that the presence of PP-*g*-MA improves the fiber wetting [25].

In light of the foregoing consideration, the significant improvement of the flexural parameters such as modulus and strength detected for the specimens containing the coupling agent is reasonable. In particular, the flexural strength of PPC/Basalt specimens was about twice the value estimated for composites based on neat PP.

**Figure 3:**
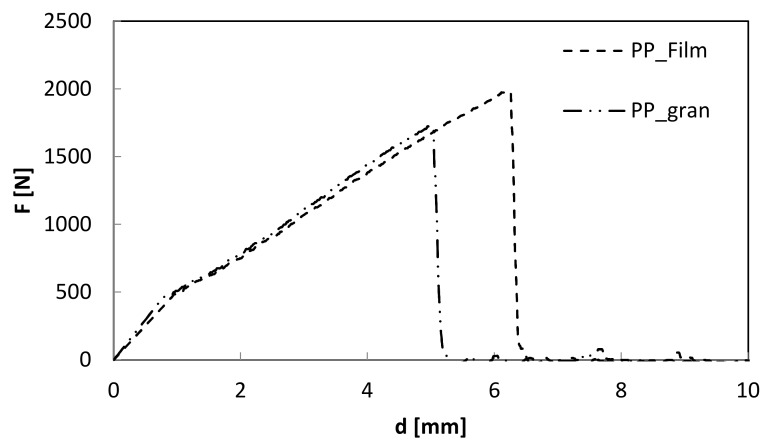
Load–displacement curves at penetration of PP granule and PP film-based samples.

Figure 3 compares reference materials in terms of load–displacement curves at penetration. From the curves, it is possible to note the higher maximum load, F_max_ and the corresponding displacement value, d, for the PP film-based specimens (see Table 2).

**Figure 4:**
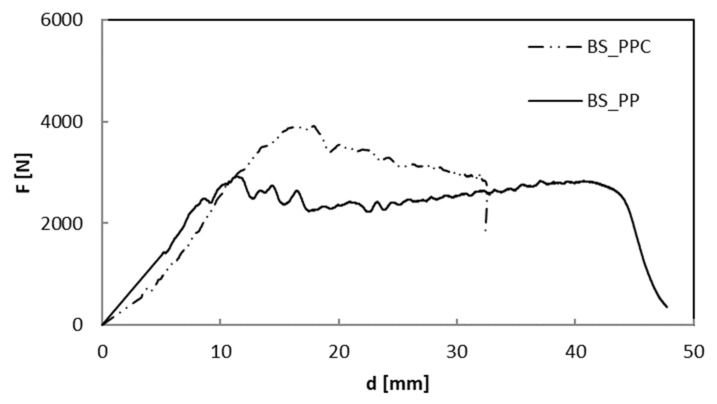
Load–displacement curves up to penetration: comparison between BS_PP and BS_PPC.

In Figure 4, the impact curves at the penetration of composite materials are reported. 

Remembering that the slope of the first linear part of the curve is an index of the impact rigidity of the system tested, the compatibilized system showed a slightly lower impact rigidity compared to the PP/Basalt one.

However, as also highlighted by the average data summarized in Table 3, it is capable of supporting higher loads (F_max_) before the reinforcement breaks recording a higher deflection, d, in correspondence of the maximum load.

**Figure 5:**
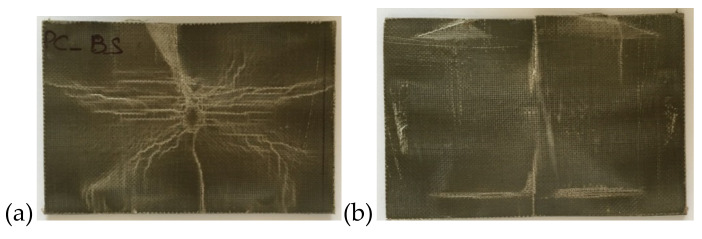
Impacted PPC basalt samples at penetration: (**a**) Front; (**b**) back.

**Figure 6:**
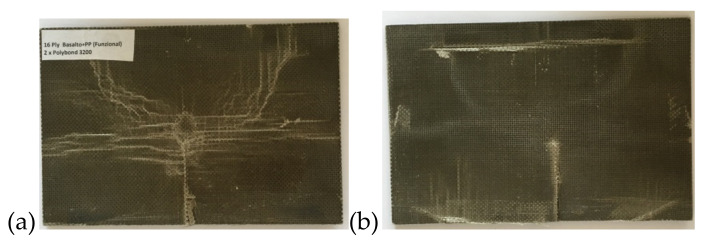
Impacted PP basalt samples at penetration: (**a**) Front; (**b**) back.

Furthermore, interestingly, in both cases, it was not possible to penetrate the panels even if a higher value of the maximum load, U_max_, is recorded for the PP/Basalt composite sample (Table 3). This behavior is highlighted in Figure 5 and Figure 6. It is evident that both the non-compatibilized and the compatibilized specimens battle to the penetration presenting only a hunching due to the impact event.

**Figure 7:**
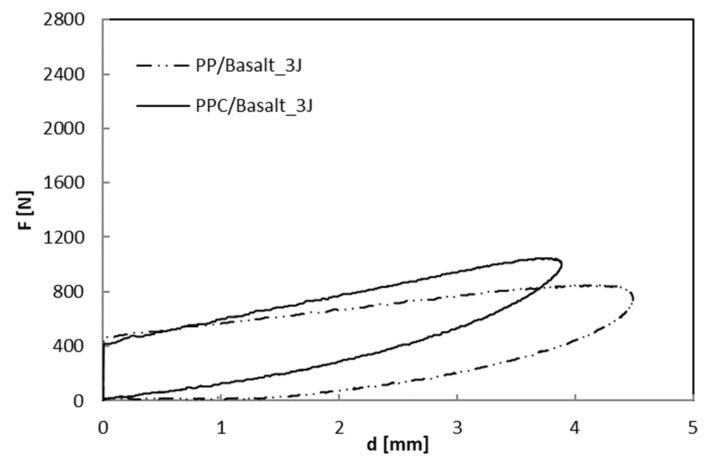
Load–displacement curves at indentation: comparison between BS_PP and BS_PPC; U = 3 J.

**Figure 8:**
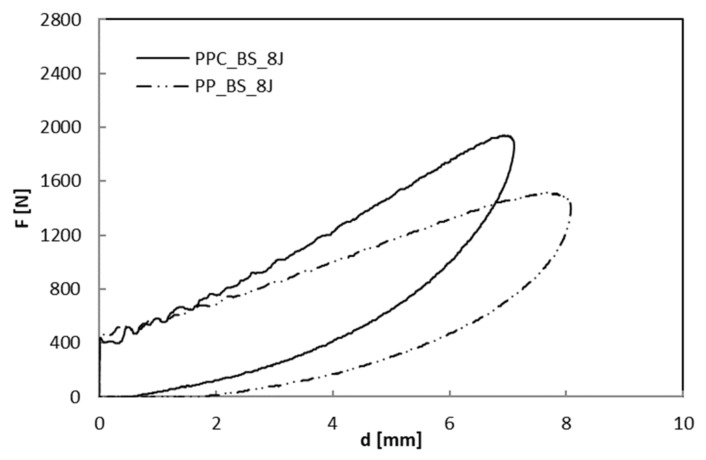
Load–displacement curves at indentation: comparison between BS_PP and BS_PPC; U = 8 J.

**Figure 9:**
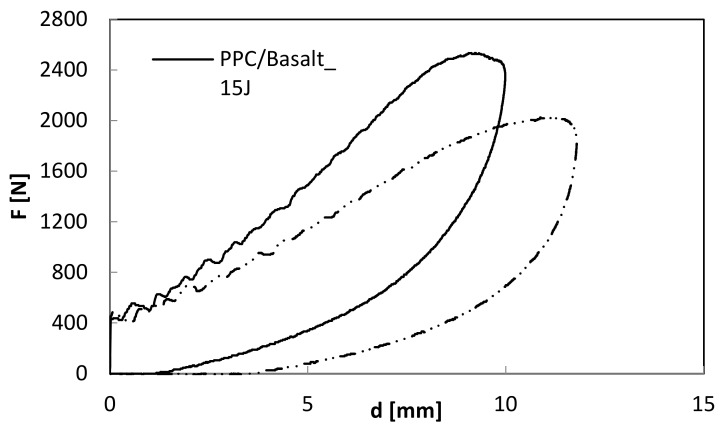
Load–displacement curves at indentation: comparison between BS_PP and BS_PPC; U = 15 J.

As far as the indentation measurements are concerned, in Figure 7, Figure 8 and Figure 9 load–displacement curves of PP and PPC samples for three impact energy levels, U, are reported. 

**Figure 10:**
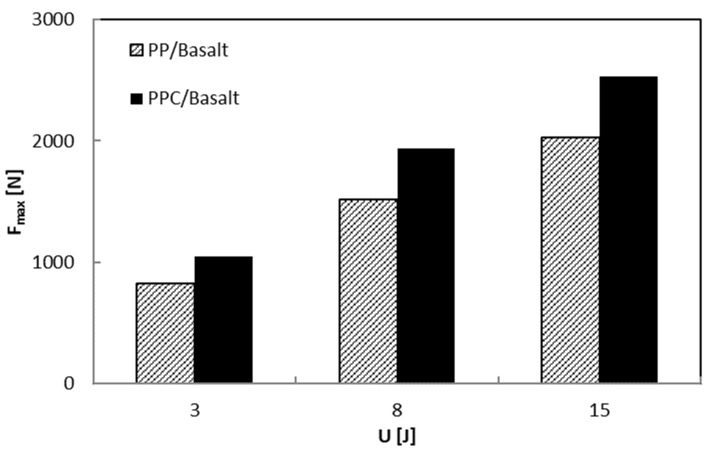
Maximum load, F_max_, versus impact energy, U.

**Figure 11:**
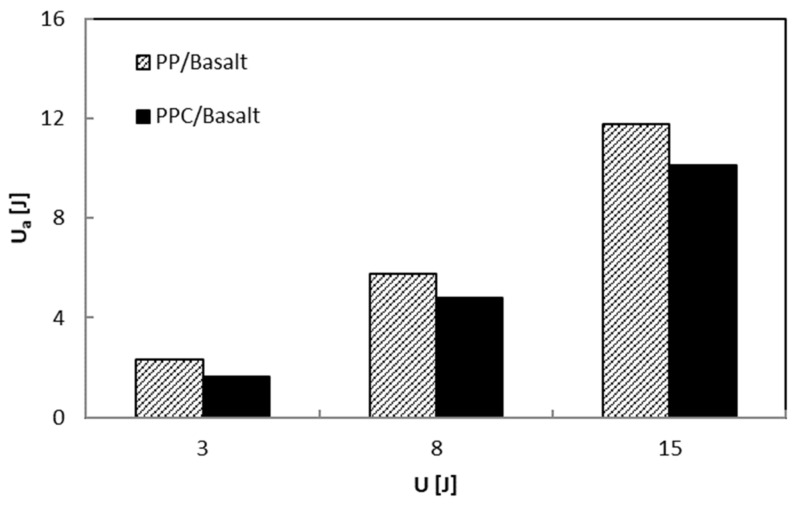
Absorbed energy, U_a_, versus impact energy, U.

Taking into account that the area enclosed in the indentation curves represents the energy absorbed by the laminate to create damage, U_a_, this parameter and the maximum impact load, F_max_, increase as the impact energy, U, increases as shown in Figure 10 and Figure 11, respectively. In particular, the U_a_ values, reflecting the extent of induced internal damage, are more significant for the PP/Basalt samples than for the PPC/Basalt ones for every tested energy indicating the occurrence of minor damage for the latter, under similar impact conditions.

**Figure 12:**
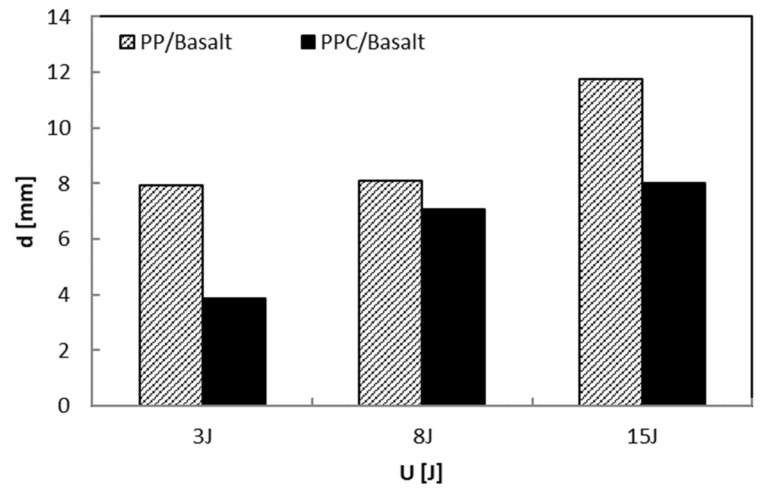
Maximum deflection, d, versus impact energy, U.

The increasing load usually results in a greater maximum deflection (Figure 12). The presence of the compatibilizer does not change this effect even if it gives rise to minor deflections to indicate a small amount of energy spent on bending. For the PP/Basalt system, the trend of the deflection has a stronger rise after U = 8 J, lowering the bending. The latter results in a greater amount of energy absorbed (see Figure 11), indicating global higher damage in the PP/Basalt system.

**Figure 13:**
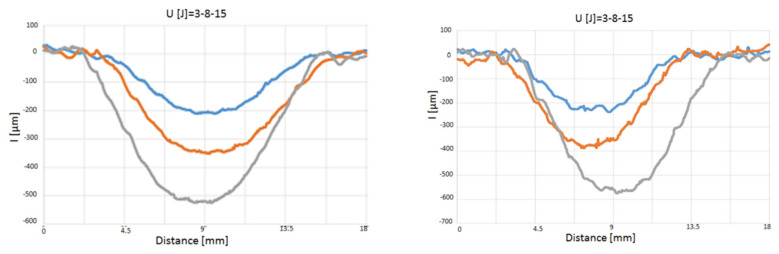
BS-PP and BS-PPC Indentation profiles.

The last assertion is confirmed by measurements of the indentation depth representing the footprint impress by the impactor on the impacted side of the sample (residual plastic deformation) shown in Figure 13. The results demonstrated that this parameter increases at the increasing of the impact energy, U, for both types of basalt composite samples.

**Figure 14:**
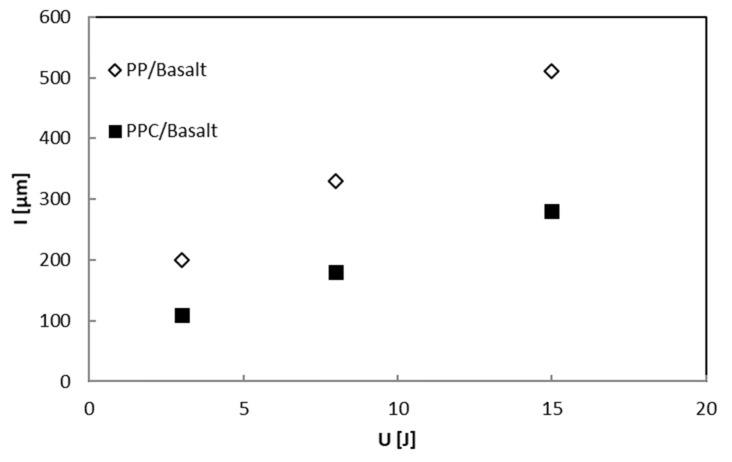
PP/Basalt and PPC/Basalt Indentation depth, I, versus the impact energy, U.

Figure 14 shows the trend of the indentation depth, I, as a function of the impact energy, U. It is possible to note that the indentation, I, measured on the basalt PP samples is higher than that shown by the PPC ones on the entire range of impact energies examined: it was an expected effect since the best interfacial adhesion prevents plastic deformation of the matrix.

## 4. Conclusions

Polypropylene/basalt fabric composite laminates prepared by film piling and compression molding techniques were analyzed. Flexural and low-velocity impact properties were evaluated, taking neat PP plates as references and considering effects related to matrix modification with a pre-optimized amount of a maleic anhydride grafted PP as a coupling agent. Mechanical results demonstrated the preliminary change of the matrix to enhance the interfacial adhesion leads to samples with improved flexural performances especially the strength and ability to withstand higher impact loading with respect to neat PP based composite laminates. Premised that for both investigated systems, no penetration seems to occur, the more significant matrix-reinforcement interaction for compatibilized composite laminates make them less prone to plastic deformations as also evidenced by the indentation measurements. The indentation, I, recorded for the basalt PP samples is higher than the PPC ones confirming the higher absorbed energy, U_a_ that denotes greater damage due to the impact tests.

## Figures and Tables

**Table 1:** Flexural stress–strain results.

Sample	Flexural Modulus (MPa)	Flexural Strength (MPa)
PP/Basalt	13790 ± 820	39.9 ± 2.0
PPC/Basalt	15940 ± 788	81.1 ± 2.2

**Table 2:** Impact parameters at penetration for matrix.

Matrix	F_max_ (N)	d (mm)
PP_gran	1689	4.99
PP_film	1964	6.25

**Table 3:** Impact at penetration parameters for both composite systems.

Type	F_max_ (N)	d (mm)	U_max_ (J)
BS/PP	2919.58	14.88	102.88
BS/PPC	3918.91	17.12	84.34
